# A linguistic approach to the psychosis continuum: (dis)similarities and (dis)continuities in how clinical and non-clinical voice-hearers talk about their voices

**DOI:** 10.1080/13546805.2020.1842727

**Published:** 2020-11-06

**Authors:** Luke C. Collins, Elena Semino, Zsófia Demjén, Andrew Hardie, Peter Moseley, Angela Woods, Ben Alderson-Day

**Affiliations:** a ESRC Centre for Corpus Approaches to Social Science, Lancaster University, Lancaster, UK; b Institute for Education, University College London, London, UK; c Psychology Department, Northumbria University, Newcastle-Upon-Tyne, UK; d Hearing the Voice, Durham University, Durham, UK

**Keywords:** Psychosis, continuum, corpus linguistics, auditory verbal hallucinations, voice-hearing

## Abstract

**Introduction**: “Continuum” approaches to psychosis have generated reports of similarities and differences in voice-hearing in clinical and non-clinical populations at the cohort level, but not typically examined overlap or *degrees* of difference between groups.

**Methods**: We used a computer-aided linguistic approach to explore reports of voice-hearing by a clinical group (Early Intervention in Psychosis service-users; *N *= 40) and a non-clinical group (spiritualists; *N *= 27). We identify semantic categories of terms statistically overused by one group compared with the other, and by each group compared to a control sample of non-voice-hearing interview data (log likelihood (LL) value 6.63+=*p *< .01; effect size measure: log ratio 1.0+). We consider whether individual values support a continuum model.

**Results**: Notwithstanding significant cohort-level differences, there was considerable continuity in language use. Reports of negative affect were prominent in both groups (*p *< .01, log ratio: 1.12+). Challenges of cognitive control were also evident in both cohorts, with references to “disengagement” accentuated in service-users (*p *< .01, log ratio: 1.14+).

**Conclusion**: A corpus linguistic approach to voice-hearing provides new evidence of differences between clinical and non-clinical groups. Variability at the individual level provides substantial evidence of continuity with implications for cognitive mechanisms underlying voice-hearing.

## Introduction

In clinical settings, hearing voices (or auditory verbal hallucinations, AVH) is typically associated with a diagnosis of schizophrenia (Bauer et al., 2011; Sartorius et al., 1986), though hearing voices can also occur in other disorders such as bipolar disorder (Toh et al., 2016) and post-traumatic stress disorder (Brewin & Patel, 2010), where they can be distressing, debilitating, and often treatment-resistant experiences. Consistent with a “continuum” approach to understanding psychotic experiences (Van Os et al., 2000), however, voices are also reported by individuals without a need for care, with a number of studies contrasting voice-hearing in clinical and non-clinical voice-hearers in terms of phenomenology (Powers, Mathys, et al., 2017; Sommer et al., 2010), cognition (Daalman et al., 2013), or brain activity (Diederen et al., 2012; Powers, Mathys, et al., 2017). The continuum approach suggests that such “non-clinical voice-hearers” (NCVHs) are situated on a continuous dimension between non-voice-hearing individuals and voice-hearers with a psychiatric diagnosis (Baumeister et al., 2017). However, there have been concerns regarding the validity of the continuum approach (David, 2010), with suggestions that multiple continua might be involved (Johns et al., 2014) and that continuities in experience should not be taken to necessarily reflect continuity in terms of underlying neurocognitive mechanisms (Waters & Fernyhough, 2019).

A systematic review of studies of voice-hearing in NCVHs (Baumeister et al., 2017) demonstrated that the research cited in debates around a “psychosis continuum” has typically relied on quantitative measures and the identification of significant differences (or not) between populations. For example, various studies have reported similarities between clinical voice-hearers (CVHs) and NCVHs in terms of the acoustic characteristics, loudness, location, number of voices heard, and personification of voices (De Boer et al., 2016; Powers, Kelley et al., 2017). Nevertheless, the two groups have also been shown to differ on the interpretation of and beliefs about the experiences, perceived ability to control them, and affective responses to them (Daalman et al., 2011; Johns et al., 2014; Powers et al., 2017; Woods & Wilkinson, 2017). Indeed, the most prominent reported differences between voice-hearing in CVHs and NCVHs relate to the emotional valence of the content, with the latter reporting more negative experiences, predicting the presence of a psychotic disorder in 88% of the participants (Daalman et al., 2011).

However, studies reporting group similarities and/or differences do not allow investigation of overlap between and within groups or degrees of difference on any given scale. This approach to “difference” could be seen as actually reinforcing cohort boundaries, while shedding little light on what true continuity would look like for people who hear voices. David (2010) has called upon researchers interested in the “psychosis continuum” to consider the variability of the phenomena within or between individuals, and to “define in advance what would constitute evidence of discontinuity” (p. 1940). This demands an approach to measurement that allows researchers to evaluate discrepancies between (reported) experiences as incremental or discontinuous.

While linguistic choices in reports of voice-hearing may not straightforwardly reflect phenomenology, we suggest that the systematic analysis of similarities/differences and continuities/discontinuities in linguistic expression is an essential complement to other approaches to the psychosis continuum. Stanghellini et al. (2012) argue that experiences of voice-hearing cannot be reliably assessed without some characterisation of the phenomenal *quality* of the experience, with one systematic review revealing a lack of qualitative studies of voice-hearing (Upthegrove et al., 2016). Those studies that do have a qualitative component typically involve semi-structured interviews and have demonstrated how some of the complexity of participants’ sense-making and coping strategies can be captured in this way (Fenekou & Georgaca, 2010; Milligan et al., 2013; Woods et al., 2015). The exploratory aspect of semi-structured interviews can “yield insights into what people who hear voices themselves regard as most important” (Woods et al., 2015, p. 33). This is consistent with a language-based approach to personal accounts, whereby the language choices made by participants are understood as reflecting the “relative importance of some narrative units as opposed to others” (Labov & Waletzky, [1967] 1997, p. 32). Linguistics has been used recently to examine differing use of first- and second-person constructions in people with schizophrenia (Tovar et al., 2019), and to compare differences in the linguistic structure of voice “utterances” in clinical and non-clinical voice-hearing (De Boer et al., 2016). However, this work has largely focused on syntactic and grammatical differences that may reflect underlying deficit or disorder, rather than using linguistics to illustrate and elucidate experiential qualities of voice-hearing (but see Demjén et al., 2019; Demjén et al., 2020).

In this paper, we used a computer-aided linguistic approach to explore similarities/differences and continuities/discontinuities in the language used in reports of experiences of voice-hearing by CVHs and NCVHs. We investigated the extent to which an analysis of the language used by different groups when describing voice-hearing experiences supports or challenges the notion of a psychosis continuum (or continua), and explored the degree of overlap between groups in terms of the language used. We applied well-established analytical techniques from Corpus Linguistics (McEnery & Hardie, 2012) to the transcripts of two sets of phenomenological interviews with (a) a group of NCVHs: “spiritualist” participants who reported regularly hearing voices (“clairaudience”); and (b) 40 Early Intervention in Psychosis (EIP) service-users reporting regular AVHs. In prior research on non-clinical voice-hearing, opportunity sampling has very often involved recruiting from spiritualist churches and similar spiritual communities, which will often identify individuals with benign experiences of voice-hearing from a young age (Alderson-Day et al., 2017; Peters et al., 2016; Powers, Kelley, et al., 2017; Sommer et al., 2010). Sampling largely from spiritual/spiritualist groups may run the risk of missing community-specific differences in unusual experiences (Luhrmann, 2017; Woods & Wilkinson, 2017), but experimental evidence has highlighted overlapping cognitive and neural processes between these groups and clinical participants (Diederen et al., 2012; Powers, Mathys, et al., 2017), lending some confidence to the comparison. Here – consistent with prior research – we use a sample of spiritualist voice-hearers as a proxy example of non-clinical voice-hearing more generally.

We employed an existing software tool for the semantic annotation of texts to analyse the frequencies of words pertaining to previously discussed aspects of voice-hearing (affect, control, meaning-making, and sensory characteristics), and to compare the groups both to each other and to a “reference corpus” of more general interview talk. We further investigated the dispersion of references to each aspect at the individual level across and within groups, to explore the degree of overlap between the two groups.

## Methods

### Data

We examined semi-structured interview data in which participants (*N* = 67) discussed their voice-hearing experiences with one of two interviewers trained in clinical and phenomenological interviewing. The samples – which had been collected previously as part of Durham University’s *Hearing the Voice* project (Alderson-Day et al., 2020) – consisted of 40 CVHs using Early Intervention for Psychosis services (*N* female = 17, mean age = 28.7, *SD* = 9.96, mean time in services = 114.1 days, *SD* = 64.8) and 27 NCVHs (*N* female = 19, mean age = 58.0, *SD* = 12.0): “spiritualist” participants reporting regular “clairaudient” voice hearing experiences, who had been recruited via the Spiritualist National Union. Developed from Woods et al. (2015), the interview builds on eight open-ended questions (with subsequent prompts for comparison and elaboration) used to elicit discussion (see *Supplementary Materials*). Interviews typically lasted one hour (range: 24–124 min) and were transcribed verbatim according to a standard protocol. Users of Early Intervention in Psychosis (EIP) services were invited to take part if they (i) were aged 16–65^1^; (ii) heard voices at least once a week for a month^2^; (iii) were fluent English speakers; (iv) had normal or corrected-to-normal vision; and, (v) were in the first nine months of using EIP services. Exclusion criteria for service-users were: (i) the presence of a suspected duration of untreated psychosis longer than five years; (ii) any neurological diagnoses; or (iii) having a hearing impairment that required the use of hearing aids. Spiritualists were invited to take part if they were (i) aged 18–75; (ii) reported hearing voices at least once per month for at least the last three months; and (iii) had normal or corrected-to-normal vision. Exclusion criteria for the spiritualists were having had contact with services for mental health issues or a psychiatric or neurological diagnosis; a recent history of drug or alcohol abuse (i.e., in the previous 3 months)^3^; or a diagnosed hearing impairment. All participants were asked the same interview questions (allowing for distinct elaborations relating to each participant’s experiences). Participants provided written consent, including for the anonymised reproduction of direct quotes from their interviews. All procedures were approved by either a local NHS Research Ethics Committee or university research ethics committee.

### Corpus linguistic methodology

Corpus Linguistics refers to a range of approaches that use tailor-made software tools to study patterns of language use in collections of texts, or “corpora” that are typically too large to be analysed manually (McEnery & Hardie, 2012). Here we conducted: (1) “keyness” analyses, to identify statistically significant differences between the two sets of interviews, and between each set of interviews and a “reference corpus” in terms of “semantic domains” (areas of meaning); and (2) “dispersion” analyses, by plotting the relative frequency values for key areas of meaning for each participant, to determine whether the distribution of these linguistic features across individuals supported the concept of a “continuum”. Only the participants’ contributions to the interviews were subject to linguistic analysis, but the interviewer’s questions were considered to contextualise participants’ answers as relevant.

Keyness analysis is used to compare the relative frequencies of words, grammatical categories or semantic domains across two datasets (corpora) to determine the language features that occur with unusually high frequency (“key” or “overused”) in one dataset compared to another. “Key” features are taken to be characteristic of that dataset. We employed the UCREL Semantic Analysis System (USAS) tagger in the online corpus software Wmatrix (Rayson, 2008) to semantically annotate our data and carry out keyness analyses at the level of semantic domains. The USAS tagger automatically allocates the content of a corpus into a pre-determined set of categories that group together words with similar semantic meaning and this categorisation system has been applied across a range of studies (Rayson, 2008). It includes 21 general semantic domains (e.g., “Emotion”) and 232 more specific sub-domains (e.g., “Sad” as a sub-domain of “Emotion”) (see *Supplementary Materials*). The general categories are indicated by a letter and the sub-domains are numbered. Further subdomains indicate contrasting valence (e.g., “E4.1+ Happy” versus “E4.1- Sad”) and degree, as with the category “X3.2+”, which would contain the word “loud”, compared with “X3.2++”, which would contain “louder” (NB: In the Results and Discussion sections, we only include the alphanumeric codes when they are needed to distinguish between different subdomains). When applied to conversational data, the tool has a reported level of accuracy of approximately 91% (Rayson, 2008) and referring to the original interview context allows us to check for any tagging errors. As participants might use different words for similar concepts (e.g., “frightened” or “scared”), this approach allows us to identify recurrent themes in the data.

We relied upon two statistical measures to identify “key” semantic domains:

- Log Likelihood (LL) – a measure of statistical significance sensitive to the size of the evidence that a difference exists (Dunning, 1993). Our findings meet a minimum confidence measure of 6.63, equivalent to *p* < .01.
- Log ratio – a measure of effect size, i.e., the binary log of the ratio of relative frequencies in the two datasets (http://cass.lancs.ac.uk/log-ratio-an-informal-introduction/). This indicates the degree of difference between relative frequency values. To focus on the semantic domains that demonstrated the most difference, we set a minimum threshold of 1.0, which indicates that the relative frequency for a dataset is at least twice that of the comparison data.^4^

We carried out two keyness comparisons:

1. a direct comparison of the interview responses of the two cohorts, identifying the semantic domains that are statistically overused by CVHs compared to NCVHs and vice versa. These key semantic domains reflect the largest *differences* in relative frequencies between the groups, reported in terms of effect size;
2. a comparison of each cohort’s interviews against a corpus of oral history interviews taken from the British National Corpus and consisting of 777,132 words (Aston & Burnard, 1998). This reference corpus matched our own data in terms of genre and style, but was more general in terms of topics discussed.

Semantic domains that were overused by only one group – in comparison with the reference corpus – provided another indication of difference. Semantic domains that were overused by *both* groups – in comparison with the reference corpus – indicate potential areas of *similarity* in the language use of NCVHs and CVHs. In such cases, examining effect size values makes it possible to consider *degrees* of similarity between the two groups, with respect to more general language use.

Semantic domains that emerged as key in these comparisons were grouped according to the aspects of the voice-hearing experience that they were used to describe (see Table 1). These groupings were arrived at inductively by exploring how the constituent terms in each domain were used in context, and bringing together domains that were related in their usage. For example, the following semantic domains were used by participants to express negative emotions in relation to voice-hearing experiences, and were therefore subsumed under the grouping *Negative affect* (e.g., “And then I get very upset”):

- E2- Dislike (e.g., including “hate”, “hates”)
- E4.1- Sad (e.g., “upset”, “sad”, “grief”)
- E5- Fear/shock (e.g., “scared”, “scary”, “panic”, “fear”)
- E6- Worry (e.g., “anxiety”, “stress”, “distressing”).

**Table 1.** Themes and groupings of key semantic domains.

Theme	Grouping	Constituent semantic domains	Constituent words	Compared with reference corpus (overused by:)	Log Ratio	Direct comparison (overused by:)	Log Ratio	Extracts relating to AVHs (Terms in the semantic domain are underlined)
***1. Affect***	*Negative affect*	E2- Dislike	hate, hates	CVHs	1.28	CVHs	1.23	**CVH-31:** “So hearing these things is a lot more scary. It makes me quite depressed” **NCVH-23: “**I think when you’re down, you’re depressed and your mind’s not right, you see bad things”
		E3-- Violent/Angry	angrier	CVHs	4.92			
		E4.1- Sad	upset, sad, grief	NCVHs	1.12			
				CVHs	1.95			
		E5- Fear/shock	scared, scary, panic, fear	NCVHs	1.42	CVHs	1.11	
				CVHs	2.52			
		E6- Worry	anxiety, stress, distressing	CVHs	1.56	CVHs	1.25	
	*Negative evaluation of self*	I1.3- Cheap	worthless			CVHs	5.46	**CVH-11: “**I feel like an idiot” **NCVH-24: “**It sounds like I’m a nutter!”
		S1.2.5- Weak	vulnerable, weak	CVHs	2.44	CVHs	1.73	
		S1.2.6- Foolish	stupid	CVHs	1.86	CVHs	1.00	
		X9.1- Inability/unintelligence	idiot, unable			CVHs	1.79	
	*Positive affect*	E4.2+ Content	proud, pleased			NCVHs	1.38	**CVH-40: “**you’re glad to see them” [the voices] **NCVH-08: “**it’s a feeling of contentment”
		E6+ Confident	confidence, reassurance			NCVHs	1.08	
***2. Control***	*Disengagement*	A1.9 Avoiding	avoid, leave me alone			CVHs	1.14	**CVH-25:** “I thought, just go away, leave us alone, then it went away” **NCVH-02:** “And I was saying to her, please leave me alone”
		Q2.1- Speech: Not communicating	shut up, keep quiet	CVHs	2.85	CVHs	2.41	
		X5.1- Inattentive	ignore, distract	NCVHs	2.35	CVHs	2.47	
				CVHs	4.82			
	*Command over (the voices)*	A1.5.1 Using	use, using			NCVHs	1.34	**CVH-30:** “I tried to show my Dad writing that wasn’t there” **NCVH-02:** “it is usual for the medium to give, I would say at least four messages”
		A10+ Open; Finding; Showing	find, open, show, pinpoint			NCVHs	1.06	
		A9- Giving	give, giving			NCVHs	1.86	
	*Developing understanding*	P1 Education in general	teacher, training, students			NCVHs	1.13	**CVH-05:** “I knew stuff was wrong, so I would learn tips like externalisation” **NCVH-01:** “within the last ten years, I’ve probably learnt to fine-tune it better”
		X2.3+ Learning	learn, learning			NCVHs	1.32	
		X9.1 Ability and intelligence	faculty, calibre			NCVHs	3.96	
***3. Meaning-making***		A1.2+ Suitable	relevant, appropriate			NCVHs	1.72	**CVH-27:** “it’s just random what they say, it’s never anything important or whatever” **NCVH-13:** “the message will always be different, there will be different evidence given”
		A1.6 Concrete/Abstract	philosophical, abstract	NCVHs	1.71	NCVHs	5.79	
		A11.1+ Important	important, main			NCVHs	1.61	
		A5.2+ Evaluation: True	evidence, prove			NCVHs	1.30	
		Q1.1 Linguistic Actions, States and Processes; Communication	message(s), means	NCVHs	1.70	NCVHs	1.66	
***4. Sensory Input***		X3.2 Sensory: Sound	hear, sounds, listen	NCVHs	2.26			**CVH-40:** “the more I’ve listened to them they’ve got louder and they know they’re being heard I think” **NCVH-21:** “I hear the spirit world in my inner voice”
				CVHs	3.15			
		X3.2- Sound: Quiet	quiet, deaf, muffled, silence	NCVHs	1.46			
				CVHs	1.16			
		X3.2-- Sound: Quiet	quieter	NCVHs	3.71			
				CVHs	4.50			
	*Loudness*	X3.2+ Sound: Loud	loud	NCVHs	2.63	CVHs	1.33	**CVH-26:** “If I’m having a particularly anxious day, it gets, it feels like it gets louder and louder and louder” **NCVH-10:** “the louder it is for me, the more urgent it is to get that across”
				CVHs	3.96			
		X3.2++ Sound: Loud	louder	NCVHs	4.29	CVHs	3.24	
				CVHs	7.53			
	*Strength*	S1.2.5+ Tough/strong	strong, strengths			NCVHs	1.07	**CVH-31:** “when I feel anxious or I’m feeling down or upset, the voice comes out stronger” **NCVH-03: “**So at the times of real severe emotional upset that external voice seems to come in stronger”
		S1.2.5++ Tough/strong	stronger	NCVHs	4.43	NCVHs	1.84	
		S1.2.5+++ Tough/strong	strongest	NCVHs	4.88	NCVHs	1.96	
	*Other senses*	X3.3 Sensory: Touch	touch			CVHs	1.38	**CVH-40:** “I can feel the touch of some of the visual things I see” **NCVH-08:** “you could see her being forced to cross the room, and she touched us right there”
		X3.4+ Seen	noticed, notice	CVHs	1.37	CVHs	1.27	
		X3.5 Sensory: Smell	smell, smells	NCVHs	2.18	CVHs	1.56	
				CVHs	3.74			
	*Cognition*	X1 Psychological Actions, States and Processes	mind, trance	NCVHs	4.22	NCVHs	1.94	**CVH-23:** “I can sense that she’s going to talk” **NCVH-02:** “there could also be erm people who are not visible but you’re able to sense that they’re like round the corner”

We focused our analysis on a selection of groupings that can be subsumed under four broad themes that have been discussed in previous research on the psychosis continuum (Baumeister et al., 2017): *Affect*, *Control*, *Meaning-Making* and *Sensory Input*. In our data, the groupings that are subsumed under each of the four themes relate to the following aspects of the experience of voice-hearing (see Table 1):

- *Affect*: experiencing positive or negative emotions, and negative self-evaluations;
- *Control*: attempting to disengage from the voices, exerting command over the voices, and developing skills in voice-hearing;
- *Meaning-Making*: understanding and interpreting what the voices say;
- *Sensory Input*: sensory and cognitive processes associated with the voices.

We then examined the dispersion of the semantic domains in each of these themes across all 67 interviews. This involved plotting the aggregated relative frequencies^5^ of these domains as bar charts. Where interviews from the clinical and non-clinical cohorts alternate along the x-axis, there is potential evidence of continuity. Where there is a clear separation, either of individuals or of the cohorts along the x-axis, there is evidence of discontinuity.

## Results

Our keyness analyses generated a total of 148 overused semantic domains. The table provided in the *Supplementary Materials* contains the complete list, with effect size values (log ratio) indicating under which keyness comparison each semantic domain was found to be overused, and for which participant group. Our groupings were derived from this list.

Twelve of these groupings pertained to our four themes of *Affect*, *Control*, *Meaning-Making*, and *Sensory Input*, and will be the focus of our analysis (Table 1). These groupings accounted for 37 (25.0%) of the 148 key semantic domains. The remaining semantic domains were excluded from the current analysis because of one or more of the following reasons: they did not have a clear semantic link to other domains (e.g., “Non-resident”, which includes “homeless”); they were not relevant to our four overarching themes (e.g., “Personal Names”); they consisted of words that, in the context of our data, differed considerably in meaning (e.g., the semantic domain “E4.1+ Happy” included the word “laughing”, which referred to being amused but also ridiculed).

### Affect

The *Affect* theme included three groupings: *Negative affect, Negative evaluations of the self,* and *Positive affect.* Two domains indicating *Negative affect* were overused by both participant groups (compared to the reference corpus): “Sad” (e.g., “upset”, “grief”) and “Fear/shock” (e.g., “scared”, “panic”) (see Table 1). In both cases, effect size values indicate a larger overuse by CVHs (1.95 and 2.52 for CVHs, compared with 1.12 and 1.42 for NCVHs, respectively). When comparing the two cohorts, both these domains were found to be overused by CVHs, alongside “Dislike” (e.g., “hate”, “despise”) and “Worry” (e.g., “anxiety”, “stress”). All four semantic domains that make up *Negative evaluations of the self* were also overused by CVHs when compared to NCVHs: “Worthless”, “Weak” (e.g., “vulnerable”, “helpless”), “Foolish” (e.g., “stupid”, “ridiculous”) and “Inability/Unintelligence” (e.g., “idiot”, “unable”). Correspondingly, two semantic domains that make up the *Positive affect* grouping were found to be overused by NCVHs compared to service-users: “Content” (e.g., “proud”, “pleased”) and “Confident” (e.g., “reassurance”, “faith”).

**Figure 1.**
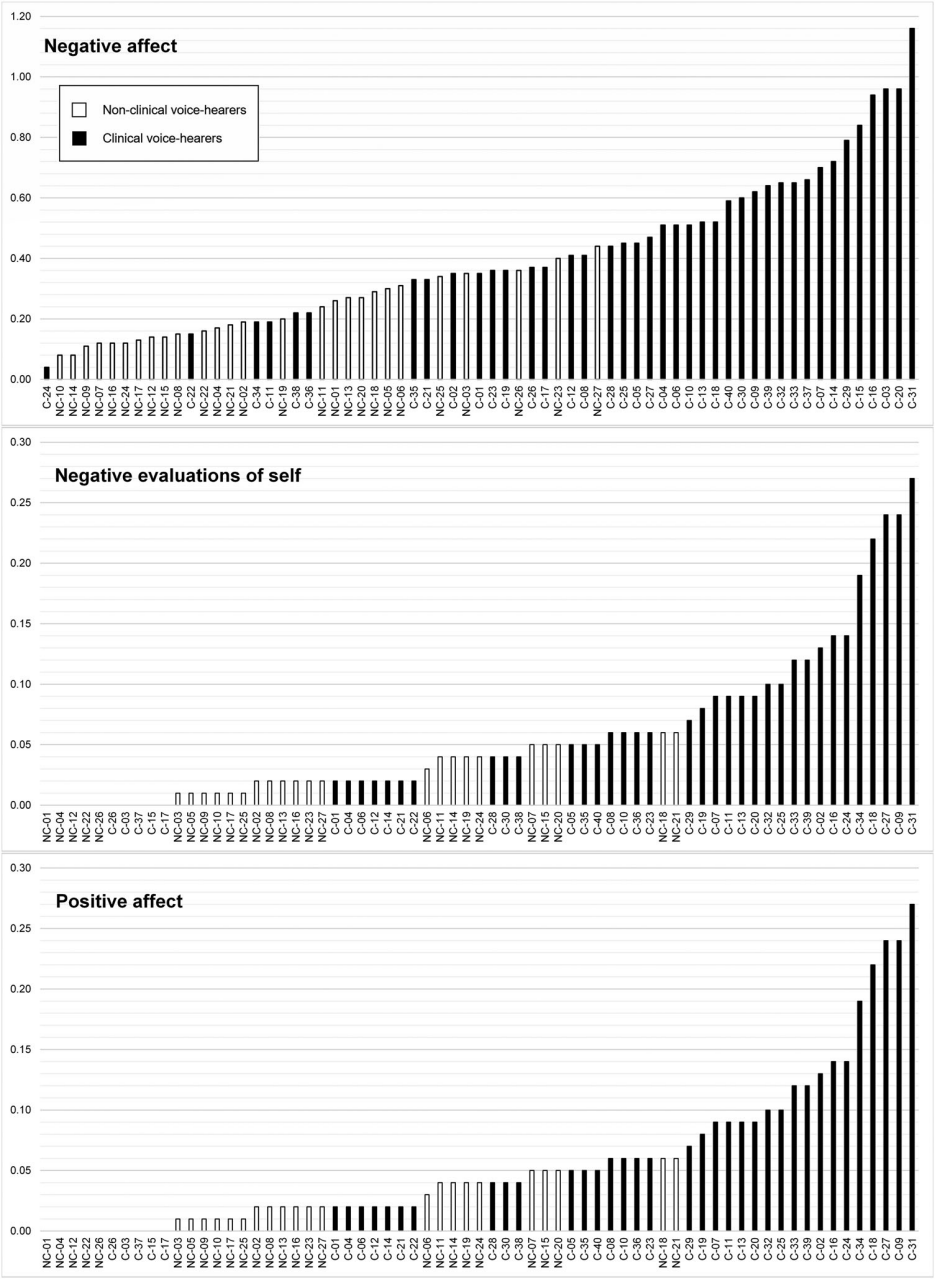
Individual relative frequency values for “Affect” terms.

The distribution of relative frequencies across individuals for each of the three *Affect* groupings is displayed in Figure 1. The overlapping of values for NCVHs (white bars) and CVHs (black bars) and incremental rise in values across participants suggests continuity. This is observed for each grouping. In the case of *Negative affect*, individual values for 44 of the 67 participants (65.7%) overlap (between 0.00 and 0.44). Similarly, 49 (73.1%) participant values for terms relating to *Negative evaluations of self* overlap. There is also overlap in the relative frequency values for *Positive affect* terms, although 16 CVHs (40%) do not use any terms in this grouping. In summary, while CVHs are more marked in their use of negative evaluative terms (for affect and the self), there is a notable overlap between the two cohorts.

### Control

The semantic domain “Inattentive” (e.g., “ignore” and “distract” in utterances such as “I’ve been trying to distract myself”) was overused by both CVHs and NCVHs compared to the reference corpus. Effect size values indicate a larger overuse in CVHs: 4.82 compared with 2.35 for NCVHs. The direct comparison showed that this semantic domain was also overused by CVHs when compared to NCVHs, along with two other domains relating to *Disengagement*: “Avoiding” (e.g., “avoided”, “leave alone”) and “Speech: Not communicating” (e.g., “shut up”, “keep quiet”) (see Table 1). In contrast, all three domains included in the grouping *Command over (the voices)* were overused by NCVHs compared to CVHs: “Using” (e.g., “choos[ing] to use my mediumship”), “Open Finding; Showing” (e.g., “open[ing] up and allowing [the voices] to come forward”), and “Giving” (e.g., “getting rid” of the voices). This was also the case for *Developing understanding*, which includes “Education”, “Learning” (e.g., “take on board”), and “Ability and intelligence” (e.g., “faculty”, “calibre”).

**Figure 2.**
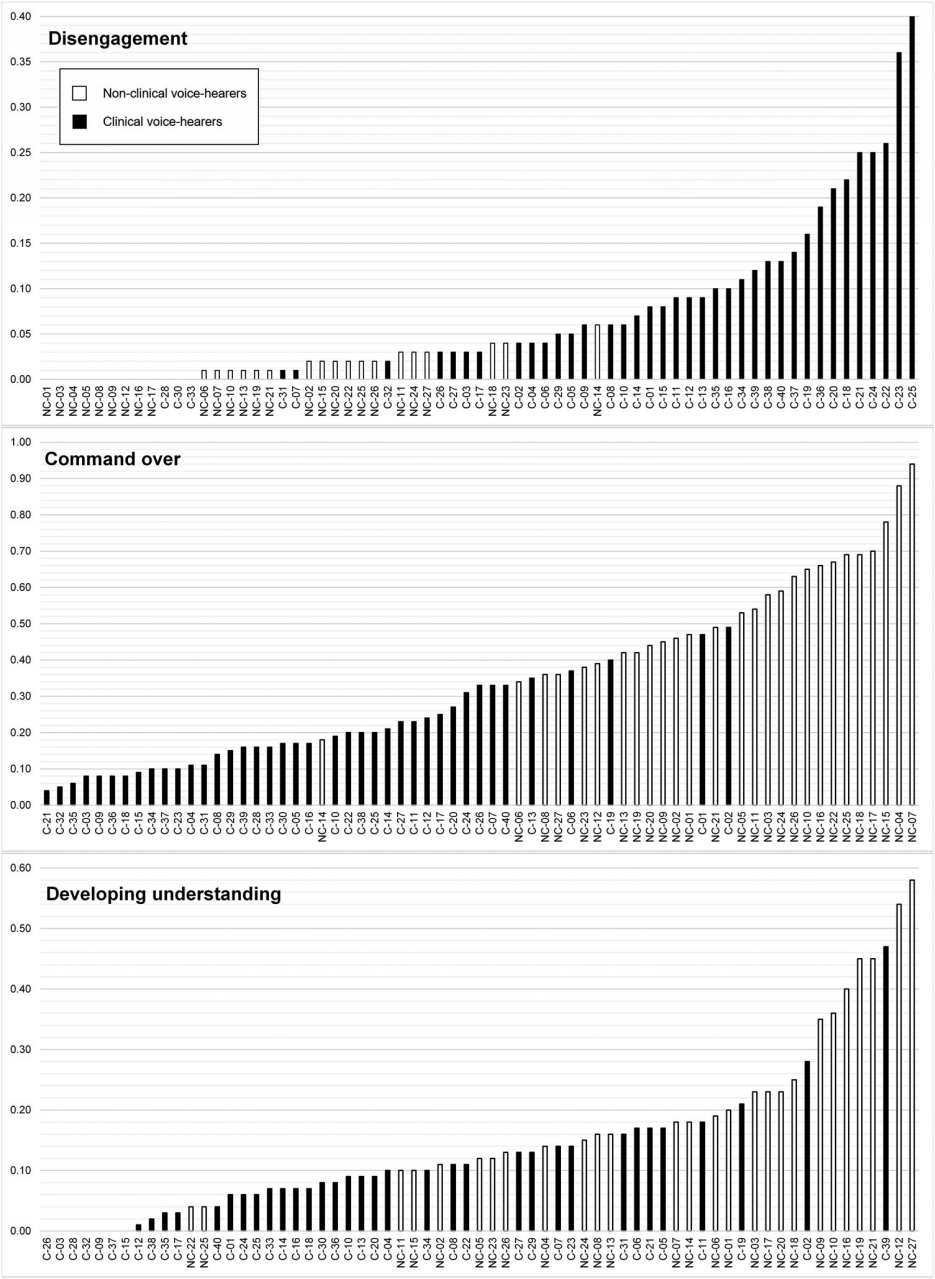
Individual relative frequency values for “Control” terms.

The dispersion of values for the *Disengagement* grouping as a whole (Figure 2) shows an overlap in the relative values for NCVHs (0.00–0.06) and CVHs (0.00–0.40), suggesting continuity. However, nine NCVHs (33% of the group) did not use any terms for this grouping, alongside three CVHs (8%). There was more evidence for continuity between the two groups for *Command over (the voices)* and *Developing understanding* (Figure 2).

### Meaning-making

The *Meaning-making* theme consists of a single grouping of five semantic domains, which were all overused by NCVHs compared to CVHs: “Suitable” (e.g., “relevant”, “appropriate”), “Concrete/Abstract” (e.g., “philosophical”, “practical”), “Important”, “Evaluation: True” (e.g., “evidence”, “prove”), and “Linguistic Actions, States and Processes; Communication” (e.g., “message”, “means”). However, an examination of these terms in context shows that both groups reflected on the “relevance” of their voice-hearing experiences to other aspects of their lives, evaluated the content of their voice-hearing experiences in terms of “prominence”, and described what the voices say as “messages”.

**Figure 3.**
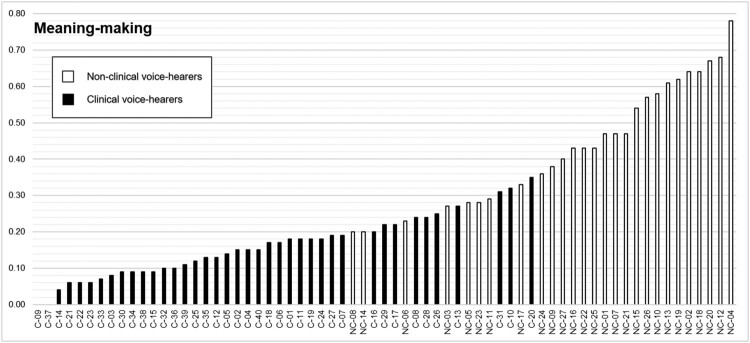
Individual relative frequency values for “Meaning-making” terms.

The dispersion of values for this theme showed an overlap in the relative frequency values for NCVHs and CVHs up to a value of 0.35 (Figure 3), suggesting some degree of continuity. The exclusive representation of NCVHs above this value – consistent with the keyness analysis – suggests that *Meaning-making* terms are particularly characteristic of the spiritualist interviews.

### Sensory input

Four semantic domains relating to sound were overused by both groups when compared with the reference corpus, reflecting the focus of some of the interview questions: “Sensory: Sound” (e.g., “hear”, “sounds”), “Sound: Quiet (X3.2-)” (e.g., “quiet”, “muffled”), “Sound: Quiet (X3.2–)” (e.g., “quieter”) and “Sound: Loud” (e.g., “noisy”, “audibly”). Effect size values show a similar degree of overuse for “Sound: Quiet (X3.2-)” and a greater overuse by CVHs for the other domains. The direct keyness comparison showed that “Sound: Loud” was overused by CVHs compared to NCVHs. In contrast, the semantic domains included under *Strength* were overused by NCVHs compared to CVHs, i.e.: “S1.2.5++ Tough/strong” (e.g., “stronger”) and “S1.2.5+++ Tough/strong” (e.g., “strongest”). These terms tended to be used in reference to the intensity of the voices, as in **“**So at the times of real severe emotional upset that external voice seems to come in stronger”.

**Figure 4.**
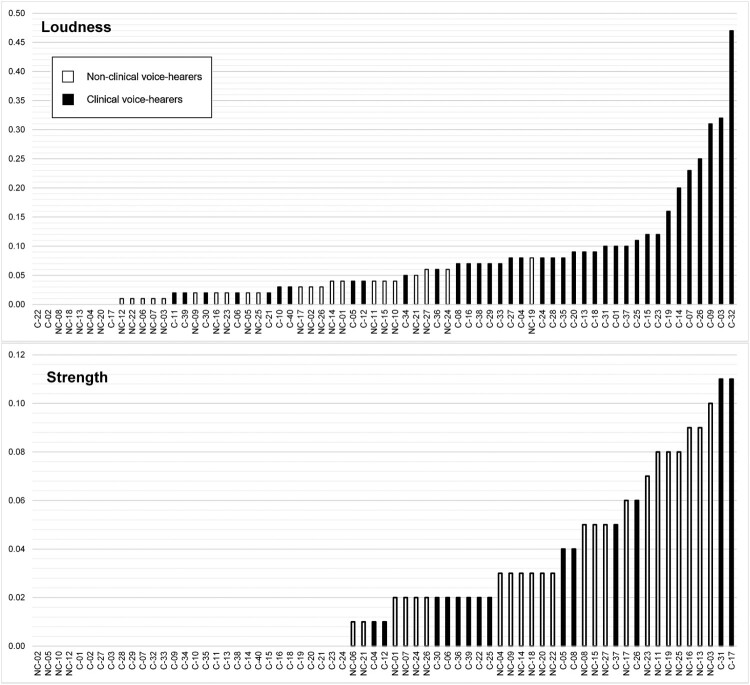
Individual relative frequency values for “Loudness” and “Strength” terms.

The dispersion of values for *Loudness* (Figure 4) shows an overlap in the relative values for NCVHs (0.00–0.08) and CVHs (0.00–0.47), suggesting some degree of continuity. However, it should be noted that no terms for this grouping were found for five NCVHs (19%) and three CVHs (8%). Similarly, while NCVHs described their sensory experiences in terms of “strength” more than CVHs, many participants in both groups did not use these terms at all.

Participants were also asked about the phenomenological aspects of their voice-hearing beyond the perception of sound. Three semantic domains grouped under *Other senses* were found to be overused by CVHs: “Sensory: Touch”, “Seen” (“noticed”, “notice”), and “Sensory: Smell”. The latter was also overused in both cohorts when compared with the reference corpus, with a higher effect size value for CVHs.

## Discussion

Debates around the “psychosis continuum” have highlighted areas of similarity and difference between clinical and non-clinical populations (Baumeister et al., 2017; Johns et al., 2014). In contrast with previous studies, our linguistic approach to the psychosis continuum makes it possible to investigate evidence of differences/similarities between individual CVHs and NCVHs (represented by Early Intervention in Psychosis service-users and spiritualists, respectively), as well as evidence of continuity/discontinuity within and across groups.

We showed that *Negative affect* terms were overused in both voice-hearing groups compared to more general interview talk (albeit to different extents), but also used much more frequently by CVHs when directly compared with NCVHs, along with terms related to *Negative evaluations of the self.* In contrast, NCVHs used more *Positive affect* terms, consistent with previous research reporting the differences in emotional valence in the responses of clinical and non-clinical groups (Daalman et al., 2011). However, our approach allowed us to extend this comparison to demonstrate that, despite these overall differences, there is also overlap and therefore some degree of continuity between the cohorts.

Likewise, consistent with previous research, our findings with respect to *Control* showed that terms related to *Disengagement* were overused by CVHs, while NCVHs more often referred to having *Command over* their voices and *Developing understanding*. This is consistent with research that has found that CVHs are more likely to try to actively ignore their voices (Kråkvik et al., 2015) and that there are differences between CVHs and NCVHs in their perceived ability to control voices (Powers et al., 2017). However, again, we observed overlap between the two cohorts in these dimensions, suggesting that control of voices may not be an “all-or-nothing” component of voice-hearing, and instead may be dimensional, with differences between CVHs and NCVHs being matters of degree rather than dichotomy. Considering that inhibitory control is one cognitive mechanism that has been associated with hallucination-proneness in both clinical and non-clinical groups (Alderson-Day et al., 2019; Paulik et al., 2007; Waters et al., 2003), these findings suggest a scenario where the spontaneity and intrusiveness of voices may naturally vary across individuals, or be flexibly tempered with training and practice (Luhrmann et al., 2019).

Similarly, previous research has also highlighted differences in the way that CVHs and NCVHs interpret their voice-hearing (Powers et al., 2017) and we found that five semantic domains relating to *Meaning-making* were significantly overused by NCVHs, compared with CVHs. This supports (now mainstream) cognitive models of voice-hearing where meta-cognitive appraisals – rather than underlying sensory atypicalities – mark out the main differences between help-seeking and healthy individuals (Peters et al., 2017).

Language about sensory components highlighted some of the subtler differences between our two cohorts. Both NCVHs and CVHs overused terms relating to the audible aspects of their voice-hearing in comparison with general interview talk, but *Loudness* terms were used much more frequently by CVHs than NCVHs, while the opposite pattern was observed with regard to use of *Strength* terms. This provides one example of the care which must be taken when assessing the phenomenology of voice-hearing experiences across such different groups, with spiritualist participants sometimes using more metaphorical than literal language to describe the sensory experience (see Luhrmann, 2017). Although both CVHs and NCVHs may describe “auditory” phenomena, this can mean many different things to different individuals (Woods et al., 2015).

For all of the aspects of the voice-hearing experience we considered, the dispersion analyses at the level of individual participants show an overlap in the relative frequencies of relevant terms for members of the two groups, supporting the notion of a continuum even in the context of a group-level difference. This offers a response to David’s (2010) call to consider the variability within or between individuals. Lawrie (2016) has argued that “[t]o overturn current practice would require convincing proof or at least some persuasive evidence that the psychosis continuum approach adds something in clinical settings” (p. 126), and so we must also consider what we learn from a continuum model. In elaborating on the similarity/difference approach to the psychosis continuum, we can begin to identify aspects of the experience where the difference between CVHs and NCVHs would appear to be a matter of degree. For example, *Meaning-making* terms are used in similar ways by members of both groups, e.g., “the voice kind of was relevant to that” (CVH) and “But they are relevant to the person” (NCVH). However, they occur much more frequently in the interviews with NCVHs. Similarly, we have shown that there is a qualitative difference in how participants discuss the intensity of their voice-hearing (i.e., with CVHs describing “loudness” and NCVHs more likely to describe “strength”) but that overall cohort differences may actually only reflect a few key individuals. In these instances, we should be wary of overstating the relationship between such a feature and clinical status, and consider whether this indicates something of the phenomenology of the individuals’ experience, or – as researchers have observed (Luhrmann, 2017) – is more reflective of a particular vocabulary, for example, that is associated with mediumship. Either way, these findings suggest that seemingly simple features of voices (e.g., loudness) are complex, and may not exist on a single dimension.

It could be considered a limitation of our work that our NCVH participants are all self-identified spiritualists, in that they may not be representative of the breadth of NCVH experiences. However, this allows us to contextualise our work among other studies of NCVHs, in which NCVH populations often comprise a high proportion of spiritualists (e.g., Sommer et al., 2010; Peters et al., 2016; Powers, Kelley, et al., 2017). Spiritualism and non-clinical status are also likely to be related – for example, due to positive appraisals of voices and expectations regarding controllability of the experience that form part of the spiritualist worldview (Peters et al., 2017) – so it is difficult to characterise a participant as either one or the other. This highlights that more work is needed to understand the interaction between spiritualism and clinical status (e.g., looking at both clinical and non-clinical spiritualist participants) and how this compares to wider varieties of non-clinical voice-hearing (Luhrmann et al., 2019). In that sense, our work provides some valuable insights into the NCVH spiritualist experience, and a basis for further exploration of language use among “healthy” voice-hearers.

Since the recruitment of CVHs targeted those newly engaging with services, there are also likely to be discrepancies between the two groups regarding the length of time they have been hearing voices (and subsequently, the development of coping strategies, for example). Similarly, the mean ages of the NCVHs (58.0) and CVHs (28.7) could have implications for how voice-hearing is experienced and reported by participants. The participant groups – on average – belong to the same “life stage” (Labov, 2001) and so we would expect that individual differences would have more of an influence on language use than age. Differences according to such participant demographics (such as age, gender, region) are likely to be minimised when the reports are aggregated and subject to statistical tests. Furthermore, our semantic domain approach allows us to group words referring to related concepts (Rayson, 2008), even if there are differences in the specific lexis according to gender, class or individual preference. Nevertheless, participants could be grouped according to more discrete age and/or duration of voice-hearing categories to investigate any potential effects on language use and analyses of individual (language) differences would need to be contextualised within individual case studies.

In addition to investigating differences in clinical and non-clinical spiritualist voice-hearers, further research should also consider the extent to which self-reports are shaped by such interpretive frameworks. While our analysis focused on semantic domains that we could relate to four key themes in the discussion of a psychosis “continuum”, some of the remaining key semantic domains (see table in *Supplementary Materials*) might be relevant to exploring other aspects pertinent to the potential need for care. For example, terms relating to alcohol and recreational drugs were overused by CVHs, while terms related to the sense of professional identity that is associated with voice-hearing were overused by NCVHs.

## Conclusion

In sum, we have shown that Corpus Linguistics offers ways of discerning patterns in the self-report of voice-hearers that can highlight (dis)similarities and (dis)continuities within and across clinical boundaries. In combining keyness analysis with an exploration of dispersion at the individual level, we have been able to report differences and some similarities between voice-hearing groups, as well as presenting measures that identify and document aspects of the voice-hearing experience that are variously “continuous” across our clinical and non-clinical groups.

## Supplementary Material

Supplemental Material

## Notes

1 This age range was determined by EIP services and restrictions for giving consent. The NCVH recruitment reflects the broadest range covered by standard research ethics procedures.

2 This requirement is informed by previous research on frequency estimates for CVHs (e.g., Kråkvik et al., 2014; Sommer et al., 2010).

3 This was to prioritise participants whose experiences are clearly not related to drugs and alcohol (following e.g., Sommer et al., 2010), though does contrast with CVH populations, which have included participants who reported alcohol or drug use (e.g., Powers, Kelley, et al., 2017).

4 A binary log ratio value of 2 indicates four times as often; log ratio of 3 indicates eight times as often, etc.

5 Given that the interviews differ in length, we normalised raw frequencies to a percentage of the individual interview data.
