# Supplementary material for: A linguistic approach to the psychosis continuum: (dis)similarities and (dis)continuities in how clinical and non-clinical voice-hearers talk about their voices
Source: Cogn Neuropsychiatry. 2020 Nov 6;25(6):447–65. doi: 10.1080/13546805.2020.1842727 (PMC7713671; doi:10.1080/13546805.2020.1842727)
Supplement: Supplemental Material [file PCNP_A_1842727_SM9505.docx]

**Supplementary Materials**

**1. The Hearing the Voice Phenomenological Interview**“For this interview I’m going to be asking some questions about the different kinds of experiences you’ve been having recently. Quite a few of the questions are going to be about hearing voices that other people cannot hear. People sometimes worry about talking about this topic: Do you have any concerns? Is it OK to ask some questions about it?”
“If you would like to stop at any point, or would prefer to talk about something else, then it is absolutely fine to do so”
“Some of my questions are going to refer to “voices”, but people can mean lots of different things by that. For some people that might involve sound, while for others it might feel more like someone or something is communicating with you. Others describe their voices as a form of telepathy, or loud thoughts. Our aim is to understand these experiences in terms that make sense for you, so please use whatever language you’re most comfortable with.”

**i. Initial voice description**“Could you try to describe to me some of the voice (or voice-like) experiences you’ve been having?”
Prompts:
- How, if at all, are these experiences different from your own thoughts?
- How, if at all, are these experiences different from hearing the voice of someone who is present in the room?
- Are there any other senses (e.g. images, tastes or smells) involved in the experience?
- Does it have a location (i.e. does it feel like you can tell where the voice is coming from)?
- Number of different voices/identities (if volunteered)
- If there is more than one voice, how do you distinguish between them? i.e. do they sound different, do they have different characters, do they say different things?
- When was the last time you had this kind of experience?

**ii. General background**“Could you tell us a bit about what life was like for you, and how you were feeling, when you first started having these experiences?”
Prompts:
- Do you remember when you first heard a voice? (establish age estimate)
- If so, can you describe it?
- Was it similar to your recent voice/voice-like experiences?
- How were things going at the time?
- When did you first seek help or treatment?

**iii. Emotions, feelings & anticipation**“How does it feel when you have the experience?”
Prompts:
- What kinds of moods or emotions are associated with your voices?
- Do you know when you are about to experience a voice? If so, how?
- Does your body feel different when you experience voices?
- Are there particular times or places when the experience is likely to occur?
- How do you feel towards your voice? Is this always the case, or does it vary?

**iv. Communication & content**“Do your experiences contain messages of any kind?”
Prompts:
- What kinds of things does the voice say?
- Are there specific words or sentences that are used? Can you give me any examples?
- Do you always understand the message (what is being said)?
- How is it expressed? (e.g. friendly, unfriendly, angry, dominant, commanding, supportive, loud, quiet)
- How can you tell how it is expressed? Is it the tone of voice, for example?
- Is the message ever spiritual or religious?

**v. Character & agency**“Does it feel as though the experiences have their own character or personality?
Prompts:
- Do you know who they are? If so, how?
- Do they remind you of anyone you know or have known?
- If so, is it the voice or what they say that reminds you of this person?
- Are they always in the same form (e.g. a voice, a vision, a presence?)
- Do they mean well?
- Do they know things that you do not?
- Are they a spiritual being?

**vi. Change over time & life impact**“Have your experiences changed at all since they first started?”
Prompts
- Has it tended to happen more or less over time?
- Over time, have you been able to influence your voices? If so, how?
- How, if at all, have your voices affected your relations with other people?

**vii. Personal meaning & interpretation**“Why do you think these experiences are happening?”
Prompts:
- What do the voices mean to you?
- Do you consider hearing voices to be a special ability or skill?
- Have they been caused by something that has happened to you, do you think?
- How do you think other people view this experience?
- Have you developed any ideas about why you have these experiences?
- Are you a religious or spiritual person?
- Have any of your family members had similar experiences?

**viii. Other experiences**“Is there anything else we haven’t talked about yet, but is an important part of your experience?”
Prompts:
- Presence of any other senses or multisensory elements (if not already covered)
- Have you also had any other experiences that you think might be linked?

**2. The UCREL Semantic Analysis System**

**A1 GENERAL AND ABSTRACT TERMS**

A1.1.1 General actions, making etc.

A1.1.2 Damaging and destroying

A1.2 Suitability

A1.3 Caution

A1.4 Chance, luck

A1.5 Use

A1.5.1 Using

A1.5.2 Usefulness

A1.6 Physical/mental

A1.7 Constraint

A1.8 Inclusion/Exclusion

A1.9 Avoiding

A2 Affect

A2.1 Affect: Modify, change

A2.2 Affect: Cause/Connected

A3 Being

A4 Classification

A4.1 Generally kinds, groups, examples

A4.2 Particular/general; detail

A5 Evaluation

A5.1 Evaluation: Good/bad

A5.2 Evaluation: True/false

A5.3 Evaluation: Accuracy

A5.4 Evaluation: Authenticity

A6 Comparing

A6.1 Comparing: Similar/different

A6.2 Comparing: Usual/unusual

A6.3 Comparing: Variety

A7 Definite (+ modals)

A8 Seem

A9 Getting and giving; possession

A10 Open/closed; Hiding/Hidden; Finding; Showing

A11 Importance

A11.1 Importance: Important

A11.2 Importance: Noticeability

A12 Easy/difficult

A13 Degree

A13.1 Degree: Non-specific

A13.2 Degree: Maximizers

A13.3 Degree: Boosters

A13.4 Degree: Approximators

A13.5 Degree: Compromisers

A13.6 Degree: Diminishers

A13.7 Degree: Minimizers

A14 Exclusivizers/particularizers

A15 Safety/Danger

**B1 Anatomy and physiology**

B2 Health and disease

B3 medicines and medical treatment

B4 Cleaning and personal care

B5 Clothes and personal belongings

**C1 Arts and crafts**

**E1 EMOTIONAL ACTIONS, STATES AND PROCESSES General**

E2 Liking

E3 Calm/Violent/Angry

E4 Happy/sad

E4.1 Happy/sad: Happy

E4.2 Happy/sad: Contentment

E5 Fear/bravery/shock

E6 Worry, concern, confident

**F1 Food**

F2 Drinks

F3 Cigarettes and drugs

F4 Farming & Horticulture

**G1 Government, Politics and elections**

G1.1 Government etc.

G1.2 Politics

G2 Crime, law and order

G2.1 Crime, law and order: Law and order

G2.2 General ethics

G3 Warfare, defence and the army; weapons

**H1 Architecture and kinds of houses and buildings**

H2 Parts of buildings

H3 Areas around or near houses

H4 Residence

H5 Furniture and household fittings

**I1 Money generally**

I1.1 Money: Affluence

I1.2 Money: Debts

I1.3 Money: Price

I2 Business

I2.1 Business: Generally

I2.2 Business: Selling

I3 Work and employment

I3.1 Work and employment: Generally

I3.2 Work and employmeny: Professionalism

I4 Industry

**K1 Entertainment generally**

K2 Music and related activities

K3 Recorded sound etc.

K4 Drama, the theatre and showbusiness

K5 Sports and games generally

K5.1 Sports

K5.2 Games

K6 Childrens games and toys

**L1 Life and living things**

L2 Living creatures generally

L3 Plants

**M1 Moving, coming and going**

M2 Putting, taking, pulling, pushing, transporting &c.

M3 Vehicles and transport on land

M4 Shipping, swimming etc.

M5 Aircraft and flying

M6 Location and direction

M7 Places

M8 Remaining/stationary

**N1 Numbers**

N2 Mathematics

N3 Measurement

N3.1 Measurement: General

N3.2 Measurement: Size

N3.3 Measurement: Distance

N3.4 Measurement: Volume

N3.5 Measurement: Weight

N3.6 Measurement: Area

N3.7 Measurement: Length & height

N3.8 Measurement: Speed

N4 Linear order

N5 Quantities

N5.1 Entirety; maximum

N5.2 Exceeding; waste

N6 Frequency etc.

**O1 Substances and materials generally**

O1.1 Substances and materials generally: Solid

O1.2 Substances and materials generally: Liquid

O1.3 Substances and materials generally: Gas

O2 Objects generally

O3 Electricity and electrical equipment

O4 Physical attributes

O4.1 General appearance and physical properties

O4.2 Judgement of appearance (pretty etc.)

O4.3 Colour and colour patterns

O4.4 Shape

O4.5 Texture

O4.6 Temperature

**P1 Education in general**

**Q1 LINGUISTIC ACTIONS, STATES AND PROCESSES; COMMUNICATION**

Q1.1 LINGUISTIC ACTIONS, STATES AND PROCESSES; COMMUNICATION

Q1.2 Paper documents and writing

Q1.3 Telecommunications

Q2 Speech acts

Q2.1 Speech etc: Communicative

Q2.2 Speech acts

Q3 Language, speech and grammar

Q4 The Media

Q4.1 The Media: Books

Q4.2 The Media: Newspapers etc.

Q4.3 The Media: TV, Radio and Cinema

**S1 SOCIAL ACTIONS, STATES AND PROCESSES**

S1.1 SOCIAL ACTIONS, STATES AND PROCESSES

S1.1.1 SOCIAL ACTIONS, STATES AND PROCESSES

S1.1.2 Reciprocity

S1.1.3 Participation

S1.1.4 Deserve etc.

S1.2 Personality traits

S1.2.1 Approachability and Friendliness

S1.2.2 Avarice

S1.2.3 Egoism

S1.2.4 Politeness

S1.2.5 Toughness; strong/weak

S1.2.6 Sensible

S2 People

S2.1 People: Female

S2.2 People: Male

S3 Relationship

S3.1 Relationship: General

S3.2 Relationship: Intimate/sexual

S4 Kin

S5 Groups and affiliation

S6 Obligation and necessity

S7 Power relationship

S7.1 Power, organizing

S7.2 Respect

S7.3 Competition

S7.4 Permission

S8 Helping/hindering

S9 Religion and the supernatural

**T1 Time**

T1.1 Time: General

T1.1.1 Time: General: Past

T1.1.2 Time: General: Present; simultaneous

T1.1.3 Time: General: Future

T1.2 Time: Momentary

T1.3 Time: Period

T2 Time: Beginning and ending

T3 Time: Old, new and young; age

T4 Time: Early/late

**W1 The universe**

W2 Light

W3 Geographical terms

W4 Weather

W5 Green issues

**X1 PSYCHOLOGICAL ACTIONS, STATES AND PROCESSES**

X2 Mental actions and processes

X2.1 Thought, belief

X2.2 Knowledge

X2.3 Learn

X2.4 Investigate, examine, test, search

X2.5 Understand

X2.6 Expect

X3 Sensory

X3.1 Sensory: Taste

X3.2 Sensory: Sound

X3.3 Sensory: Touch

X3.4 Sensory: Sight

X3.5 Sensory: Smell

X4 Mental object

X4.1 Mental object: Conceptual object

X4.2 Mental object: Means, method

X5 Attention

X5.1 Attention

X5.2 Interest/boredom/excited/energetic

X6 Deciding

X7 Wanting; planning; choosing

X8 Trying

X9 Ability

X9.1 Ability: Ability, intelligence

X9.2 Ability: Success and failure

**Y1 Science and technology in general**

Y2 Information technology and computing

**Z0 Unmatched proper noun**

Z1 Personal names

Z2 Geographical names

Z3 Other proper names

Z4 Discourse Bin

Z5 Grammatical bin

Z6 Negative

Z7 If

Z8 Pronouns etc.

Z9 Trash can

Z99 Unmatched

**3. Key semantic domains**

This table lists the individual semantic domains that were statistically overused in either the direct comparison or the comparison with the reference corpus. Effect size values indicate which comparison indicated overuse and for which participant group.

| **Semantic domain** | **Examples of constituent words** | **Direct comparison** (overuse by Log Ratio) | | **Compared with reference corpus** (overuse by Log Ratio) | |
| --- | --- | --- | --- | --- | --- |
|  |  | **Non-clinical** | **Clinical** | **Non-clinical** | **Clinical** |
| A1.1.2 Damaging and destroying | harm, break, accident |  | 1.33 |  |  |
| A1.2- Unsuitable | inappropriate, irrelevant |  |  | 3.52 |  |
| A1.2+ Suitable | relevant, appropriate | 1.72 |  |  |  |
| A1.4+ Lucky | lucky, fortunately | 2.11 |  |  |  |
| A1.5.1 Using | use, using | 1.34 |  |  |  |
| A1.5.2- Useless | pointless |  |  | 1.92 |  |
| A1.6 Concrete/Abstract | philosophical, abstract | 5.79 |  | 1.71 |  |
| A1.9 Avoiding | avoid, leave me alone |  | 1.14 |  |  |
| A10+ Open; Finding; Showing | find, open, show | 1.06 |  |  |  |
| A11.1+ Important | important, main | 1.61 |  |  |  |
| A11.2+ Noticeable | obvious, distinctive |  |  |  | 1.47 |
| A11.2- Unnoticeable | subtle, faint |  |  | 3.35 | 2.62 |
| A12+++ Easy | easiest, simplest |  |  | 2.19 |  |
| A14 Exclusivizers/Particularizers | just, only, especially |  |  |  | 1.61 |
| A2.1+ Change | happen, getting, become |  |  |  | 1.02 |
| A2.2- Unconnected | disconnected |  |  | 1.71 |  |
| A3 Being | phenomena | 4.11 |  | 4.03 |  |
| A5.1 Evaluation: Good/bad | quality, standard | 1.88 |  |  |  |
| A5.1- Evaluation: Bad | bad, terrible, poorly |  | 1.21 |  | 1.29 |
| A5.1-- Evaluation: Bad | worse |  | 4.05 |  | 2.86 |
| A5.1--- Evaluation: Bad | worst, catastrophe, disaster |  | 2.53 |  |  |
| A5.2+ Evaluation: True | evidence, prove | 1.30 |  |  |  |
| A6.2- Comparing: Unusual | weird, strange, odd, bizarre |  |  | 1.58 | 1.53 |
| A6.2+ Comparing: Usual | usually, normal, tend |  |  | 1.15 |  |
| A6.3+ Comparing: Varied | various, diverse | 1.46 |  |  |  |
| A7++ Likely | clearer |  |  | 3.88 |  |
| A7+++ Likely | definitely, conclusive |  | 1.11 | 1.40 | 2.51 |
| A8+ Seem | pop up |  | 2.67 |  | 3.38 |
| A9- Giving | give, giving | 1.86 |  |  |  |
| B1 Anatomy and physiology | head, brain, sleep, face |  |  |  | 1.08 |
| B2 Health and disease | health, toothache, coughing |  | 1.23 | 1.90 | 3.13 |
| B2- Disease | hurt, psychosis, pain |  |  |  | 1.85 |
| C1 Arts and crafts | picture, draw, design | 1.39 |  |  |  |
| E1 Emotional Actions, States and Processes General | emotional, feel, mood |  |  | 3.36 | 3.29 |
| E2- Dislike | hate, hates |  | 1.23 |  | 1.28 |
| E2+ Like | like, love, caring, enjoying |  | 1.70 |  | 2.03 |
| E2+++ Like | favourite | 3.79 |  | 1.54 |  |
| E3- Violent/Angry | aggressive, threatening |  | 1.94 |  | 1.87 |
| E3-- Violent/Angry | angrier |  |  |  | 4.92 |
| E3+ Calm | calm, relaxed, comforting |  |  | 1.16 | 1.37 |
| E4.1- Sad | upset, sad, grief |  |  | 1.12 | 1.95 |
| E4.1+ Happy | laughing, happy, funny, joke |  | 1.17 |  | 1.59 |
| E4.2+ Content | proud, pleased | 1.38 |  |  |  |
| E5- Fear/shock | scared, scary, panic, fear |  | 1.11 | 1.42 | 2.52 |
| E6- Worry | anxiety, stress, distressing |  | 1.25 |  | 1.56 |
| E6+ Confident | confidence, reassurance | 1.08 |  |  |  |
| F2++ Excessive drinking | drunk, stoned, pissed |  |  |  | 1.78 |
| F3 Smoking and non-medical drugs | smoking, cannabis, heroin |  | 1.33 |  |  |
| F4 Farming & Horticulture | field, gardener, allotment | 1.34 |  |  |  |
| G1.1 Government | country, president, minister | 1.09 |  |  |  |
| G1.2 Politics | politician, union | 2.11 |  |  |  |
| G2.1 Law and order | police, jail, punishment |  | 1.18 |  |  |
| G2.1- Crime | criminals, robbed |  | 1.84 |  |  |
| G2.2 General ethics | morals, ethics, principles | 5.18 |  | 4.78 |  |
| G2.2- Unethical | wrong, naughty, wicked |  | 1.36 |  |  |
| H4- Non-resident | homeless |  | 4.53 |  | 2.24 |
| I1.3- Cheap | worthless |  | 5.46 |  |  |
| I2.1 Business: Generally | business, company, office | 1.22 |  |  |  |
| I3.1 Work and employment: Generally | work, job, career | 1.93 |  |  |  |
| I3.2 Work and employment: Professionalism | colleague, practitioner | 3.18 |  |  |  |
| I3.2+ Professional | professional | 2.65 |  | 1.76 |  |
| I4 Industry | workshop, industrial | 1.51 |  |  |  |
| K4 Drama, the theatre and show business | scenario, script, actress | 1.71 |  |  |  |
| L1+ Alive | life, lives, alive | 1.35 |  | 2.16 |  |
| M4 Sailing, swimming, etc. | flow, swimming, surfboard | 2.38 |  |  |  |
| M5 Flying and aircraft | flying, aeroplane | 3.25 |  |  |  |
| N3.1 Measurement: General | quantify, measuring | 4.49 |  |  |  |
| N3.3-- Distance: Near | closer |  |  |  | 1.82 |
| N3.6 Measurement: Area | space, stretch | 2.25 |  |  |  |
| N3.7- Short and narrow | low, narrow |  | 1.33 |  |  |
| N3.7++ Long, tall and wide | higher, deeper, wider | 1.71 |  |  |  |
| N3.8+++ Speed: Fast | instantly |  |  | 2.93 | 3.50 |
| N5.2+ Exceed; waste | too, over, hyper |  | 1.32 |  |  |
| N6 Frequency | sometimes, twice |  |  | 2.00 | 2.53 |
| N6- Infrequent | once, rarely, occasionally |  |  | 1.02 |  |
| O1.2- Dry | dry, dried | 3.38 |  |  |  |
| O3 Electricity and electrical equipment | switch (off/on), plug, cables | 1.02 |  |  |  |
| O4.1 General appearance and physical properties | image, condition, format | 1.08 |  |  |  |
| O4.2- Judgement of appearance: Negative | nasty, horrible, awful |  | 1.24 |  |  |
| O4.5 Texture | hard, soft, scratchy |  | 1.33 |  | 1.15 |
| P1 Education in general | teacher, training, students | 1.13 |  |  |  |
| Q1.1 Linguistic Actions, States and Processes; Communication | message(s), means | 1.66 |  | 1.70 |  |
| Q1.3 Telecommunications | phone, ring, texting |  |  |  | 1.04 |
| Q2.1 Speech: Communicative | said, voice, talking |  |  | 1.29 | 1.30 |
| Q2.1- Speech: Not communicating | shut up, keep quiet |  | 2.41 |  | 2.85 |
| Q3 Language, speech and grammar | words, reading, sentence |  |  | 1.50 |  |
| Q4 The Media | edition, published, title | 8.07 |  | 4.11 |  |
| Q4.1 The Media: Books | book, library, chapter | 1.42 |  |  |  |
| Q4.2 The Media: Newspapers etc. | newspaper, magazine | 4.18 |  |  |  |
| Q4.3 The Media: TV, Radio and Cinema | telly, radio, video, movie |  |  |  | 1.04 |
| S1.1.1 Social Actions, States and Processes | contact, visit, social | 1.18 |  |  |  |
| S1.1.3+ Participating | meeting, attending | 1.30 |  |  |  |
| S1.1.4+ Deserving | deserve, worthy |  | 2.32 |  |  |
| S1.2 Personality traits | personality, temperament |  |  | 2.02 | 2.64 |
| S1.2.3+ Selfish | ego, assertive, pushy | 1.38 |  | 1.92 |  |
| S1.2.4- Impolite | rude, curse, flippant |  | 1.86 | 1.71 | 3.57 |
| S1.2.5- Weak | vulnerable, weak |  | 1.73 |  | 2.44 |
| S1.2.5+ Tough/strong | strong, strengths | 1.07 |  |  |  |
| S1.2.5++ Tough/strong | stronger | 1.84 |  | 4.43 |  |
| S1.2.5+++ Tough/strong | strongest | 1.96 |  | 4.88 |  |
| S1.2.6- Foolish | stupid |  | 1.00 |  | 1.86 |
| S3.2 Relationship: Intimacy and sex | love, embrace, sexually |  |  | 1.36 | 1.20 |
| S5- Not part of a group | personal, alone, self |  |  | 1.11 |  |
| S7.2- No respect | humiliating, degrading |  | 4.21 |  |  |
| S7.2+ Respected | respectful, admire | 2.17 |  |  |  |
| S7.3+ Competitive | competing, race | 3.60 |  |  |  |
| S9 Religion and the supernatural | spirit, church, soul, religion | 2.36 |  | 2.45 |  |
| T1.1.3 Time: Future | will, shall, future, tomorrow |  |  | 1.08 | 1.04 |
| T1.3+ Time period: long | long, ages, long-term |  | 1.05 |  |  |
| T3--- Time: New & Young | recently, lately, youngest |  | 1.02 |  | 1.35 |
| T3++ Time: Old; grown-up | older, ancient, ages |  | 1.08 |  |  |
| W1 The universe | world, universe, planet | 1.42 |  | 1.96 |  |
| W2 Light | light, shone, daylight | 1.41 |  |  |  |
| W2- Darkness | dark, darkness |  | 2.49 |  | 1.96 |
| W3 Geographical terms | earth, beach, mountain | 1.29 |  |  |  |
| X1 Psychological Actions, States and Processes | mind, trance | 1.94 |  | 4.22 | 2.28 |
| X2 Mental Actions and processes | mental(ly), memory | 1.19 |  | 2.79 | 1.61 |
| X2.1 Thought, belief | think, feel, believe |  |  | 1.29 | 1.30 |
| X2.2+ Knowledgeable | know, remember |  |  | 1.05 |  |
| X2.3+ Learning | learn, learning | 1.32 |  |  |  |
| X2.4+ Double-check | double-check |  |  | 4.71 |  |
| X2.5- Not understanding | confused, misguided |  | 1.61 |  | 1.97 |
| X2.5+ Understanding | understand, realise |  |  | 2.26 | 1.58 |
| X2.6- Unexpected | amazed, surprised | 1.75 |  |  |  |
| X3 Sensory | sensed, sensation | 1.22 |  | 3.93 | 2.71 |
| X3.2 Sensory: Sound | hear, sounds, listen |  |  | 2.26 | 3.15 |
| X3.2- Sound: Quiet | quiet, deaf, muffled, silence |  |  | 1.46 | 1.16 |
| X3.2-- Sound: Quiet | quieter |  |  | 3.71 | 4.50 |
| X3.2+ Sound: Loud | loud |  | 1.33 | 2.63 | 3.96 |
| X3.2++ Sound: Loud | louder |  | 3.24 | 4.29 | 7.53 |
| X3.3 Sensory: Touch | touch |  | 1.38 |  |  |
| X3.4+ Seen | noticed, notice |  | 1.27 |  | 1.37 |
| X3.5 Sensory: Smell | smell, smells |  | 1.56 | 2.18 | 3.74 |
| X4.1 Mental object: conceptual object | thoughts, idea, dream |  |  | 1.41 |  |
| X5.1+ Attentive | focus, concentrate |  |  | 2.59 | 2.55 |
| X5.1- Inattentive | ignore, distract |  | 2.47 | 2.35 | 4.82 |
| X5.2+ Interested/excited/energetic | exciting, fascinating | 2.68 |  | 1.50 |  |
| X5.2++ Interested/excited/energetic | manic, obsessive |  |  |  | 5.92 |
| X6- Undecided | unresolved |  |  | 3.71 |  |
| X7- Unwanted | rubbish, intrusion | 1.56 |  | 1.16 |  |
| X8+ Trying hard | trying, struggle, attempt |  | 1.32 |  | 1.86 |
| X9.1 Ability and intelligence | faculty, calibre | 3.96 |  |  |  |
| X9.1- Inability/unintelligence | idiot, unable |  | 1.79 |  |  |
| X9.2 Success and failure | Cope |  | 1.43 |  | 1.40 |
| Y1 Science and technology in general | scientist, psychologist | 1.91 |  |  |  |
| Y2 Information technology and computing | computer, e-mails, laptop |  |  |  | 1.20 |
| Z1 Personal names | Freddie, Janet, Mr Worthing | 1.09 |  |  |  |
| Z2 Geographical names | London, Irish, Water Street | 1.51 |  |  |  |
